# Letter to the Editor: Exponential Increase in COVID-19 Related Publications Compared to Other Pandemic Diseases

**DOI:** 10.5041/RMMJ.10424

**Published:** 2021-01-19

**Authors:** Antonio Liu

**Affiliations:** Neurology, Adventist Health White Memorial, Los Angeles, CA, USA

## TO THE EDITOR

I read the article by Dr Pitlik on COVID-19 with great interest.^1^ The number of publications related to the current pandemic as well as how readily available they are is yet another difference between COVID-19 and previous pandemic diseases.

When I wrote my first case report on a COVID-19 patient with meningoencephalitis in early April 2020, a literature search back then on PubMed.gov under the keyword “COVID-19” yielded a total of roughly 3,000 articles already. There has been an exponential increase in the amount of literature since. The same search today (August 22, 2020) yielded 42,693 entries. Of these, only 17 came from 2019; 42,679 are in print since January 1, 2020. Table 1 (following page) is a tabulation of the literature count for COVID-19 and selected previous pandemic diseases as outlined by Dr Pitlik.

**Table 2:** A Sample PubMed.gov search.

Search Word Used	Hits	Rough Time Frame of Articles Published	Year the Number of Articles Peaked (Number of Articles)	Estimated Rate (Number per Day)*
COVID-19	42693	2020	2020 (42679)	181
MERS in 2020	20778	2020	2020 (20778)	88
MERS before 2020	14048	Since 2003	2003 (1389)	0.98
SARS in 2020	14744	2020	2020 (14744)	63
SARS before 2020	9217	Since 1994	2004 (1229)	1
HIV	367773	Since 1985	2014 (17503)	29
H1N1	21160	Since 2000	2011 (3268)	3
Spanish Flu	1700	Since 1980	2010 (166)	0.12
Ebola	9449	Since 2000	2015 (2070)	1.3
H2N2	801	Since 1972	2015 (46)	0.05
H3N2	8097	Since 1972	2013 (547)	0.47

* Not taking into consideration any annual/monthly variation.

There is obvious similarity between “COVID-19,” “MERS,” and “SARS.” At roughly 200 new articles per day, it is a rather overwhelming amount for any scholar, physician, or administrator to digest. The number of articles per day will continue to go up—most of these articles have been published in the past 10 weeks. Besides the sheer volume, the peer review process duration has decreased by an even more astonishing rate. A normal review period of 60 days is sometimes shortened to as little as 2 or 3 days. I am sure most authors who had submitted COVID-19 related articles have experienced an “expedited publication process” to allow rapid sharing of information with the goal of providing better patient care and disease containment. Filtering misinformation will become a rather impossible task; even *New England Journal of Medicine* has retracted an article lately. It will be interesting to see if the current literature system as well as almost instantaneous access to articles will guide mankind to a more successful campaign against the current pandemic in comparison to previous ones.
